# Compound heterozygote myocilin mutations in a pedigree with high prevalence of primary open-angle glaucoma

**Published:** 2012-12-28

**Authors:** Thomas K. Young, Emmanuelle Souzeau, Lance Liu, Lisa S. Kearns, Kathryn P. Burdon, Jamie E. Craig, Jonathan B. Ruddle

**Affiliations:** 1 Centre for Eye Research Australia, University of Melbourne, Royal Victorian Eye & Ear Hospital, East Melbourne, Australia; 2 Department of Ophthalmology, Flinders University, Flinders Medical Centre, Adelaide, Australia; 3 Preston Eye Clinic, Preston, Victoria, Australia

## Abstract

**Purpose:**

To describe the phenotype of ocular hypertension and primary open-angle glaucoma in a family with individuals compound heterozygote for Gln368STOP and Thr377Met *myocilin (MYOC)* mutations.

**Methods:**

Family members of the proband underwent comprehensive ocular clinical examination and DNA sequencing for *MYOC* mutations.

**Results:**

A 34-year-old woman with marked ocular hypertension was found to carry Gln368STOP and Thr377Met *MYOC* mutations. Three other siblings carried both mutations, while one carried Gln368STOP alone. Three of five siblings had received treatment for ocular hypertension or early glaucoma, with the average age of diagnosis 28 years; one required trabeculectomy at age 27. The mother of the proband was found to be a carrier for Gln368STOP alone, which indicates that her offspring with both Gln368STOP and Thr377Met carry variants on opposing alleles.

**Conclusions:**

This pedigree is the first report with individuals compound heterozygote for the two most common glaucoma-causing *MYOC* variants. The combination of mutations manifests a more severe phenotype than either alone. Identification of gene changes associated with glaucoma within the family has enabled unaffected members to stratify their risk of future disease and institute closer monitoring and early treatment.

## Introduction

Primary open-angle glaucoma (POAG) is a complex genetic disease and one of the most common causes of visual loss worldwide. Mutations in the myocilin gene (*MYOC*, formerly known as the trabecular meshwork-induced glucocorticoid response gene) associated with POAG were discovered in 1997 and mapped to the long arm of chromosome 1 [1]. *MYOC* variants account for almost 4% of adult POAG cases, and 10% of juvenile open-angle glaucoma [2].

POAG attributable to *MYOC* gene changes is inherited in an autosomal dominant manner. Carriers tend to display elevated intraocular pressure (IOP) or open-angle glaucoma from an early age, although there may be variability in the phenotype depending on the underlying mutation [2]. Two of the most common glaucoma-causing variants of *MYOC* worldwide are Gln368STOP and Thr377Met [3]. As with most disease-causing mutations of this gene, both occur in exon 3 [4]. We describe a pedigree that to our knowledge is the first identified with individuals compound heterozygous for these *MYOC* mutations.

## Methods

Members of a family with high prevalence of glaucoma were recruited into the study. Six individuals living in Australia, comprising 4 males and 2 females were available for direct clinical examination and genotyping. Five of the patients were siblings aged 26 to 35 years. The proband’s mother age 63 was also assessed. This study was approved by the Human Research and Ethics Committee of the Royal Victorian Eye and Ear Hospital, Melbourne, and was conducted in accordance with the revised Declaration of Helsinki. Informed patient consent was obtained before enrolment.

The proband (patient V:3) was first diagnosed with ocular hypertension by her optometrist. She was referred to the Australian and New Zealand Registry of Advanced Glaucoma (ANZRAG) [5] for *MYOC* genetic testing due to her young age and strong family history of POAG. Following identification of the mutations, additional family members were ascertained and offered genetic testing through ANZRAG after providing signed consent and a blood sample.

First-degree relatives of the index case (patient V:3) lived in Australia and were available for direct assessment, except the father (patient IV:8), who was deceased. Relatives on the father’s side lived in Croatia and were not available for examination. Comprehensive medical and family history was taken by a glaucoma subspecialist ophthalmologist (JBR). Clinical details for disease-affected family members were obtained from their medical records, while unaffected individuals were invited to present for ocular examination. Data recorded included demographic details, general medical history, past ocular history, best-corrected visual acuity (BCVA), Goldmann IOP, gonioscopy, dilated fundus examination, central corneal thickness (Pachmate DGH55, DGH Technology Inc., Exton, PA), and Humphrey visual fields (HFA II, Carl Zeiss, North Ryde, Australia). For each patient, venous blood was collected by peripheral venepuncture in 2×10 ml EDTA tubes. The blood samples were stored at 2–8 °C before processing.

The criterion for ocular hypertension was IOP on repeated measurement ≥24 mmHg. A diagnosis of POAG was made in patients with glaucomatous visual field defects on a reliable Humphrey 24–2 field, including an enlarged cup-disc ratio (≥0.7) or cup-disc ratio asymmetry (≥0.2) between both eyes.

### Genotyping

The testing was performed through the National Association of Testing Authorities (NATA) accredited laboratories of the Institute of Medical and Veterinary Science (IMVS) Pathology at the Flinders Medical Centre (Bedford Park, Australia). Genomic DNA was prepared from a 200 μl sample of venous blood and extracted using an Illustra Blood Genomic Prep Mini Spin kit (GE Healthcare, Buckinghamshire, UK) according to the manufacturer’s protocols.

Each PCR was performed using 100 ng of purified genomic DNA as the template in a reaction mix containing 1.5 mM MgCl_2_, 200 μM of each deoxynucleoside triphosphate (dNTP), 1 U of Platinum Taq DNA polymerase (Invitrogen, Carlsbad, CA), 1x Platinum Taq PCR reaction buffer, and 0.5 μM of each primer (for exon 3: 3.1F: 5′-GGG CTG TCA CAT CTA CTG GC-3′, 3.1R: 5′-GCT GTA AAT GAC CCA GAG GC-3′; 3.2F: 5′-GCT GAA TAC CGA GAC AGT GAA G-3′, and 3.2R: 5′-AAC TTG GAA AGC AGT CAA AGC-3′), in a final volume of 25 μl. Samples were denatured for 5 min at 94 °C and then incubated for 15 cycles under the following conditions: 94 °C for 30 s, 61 °C for 50 s (reduced 1 °C every five cycles), and 72 °C for 60 s. The samples were then incubated for 35 cycles under the following conditions: 94 °C for 30 s, 58 °C for 50 s, and 72 °C for 60 s. The last elongation step was at 72 °C for 5 min on a Veriti thermal cycler (Life Technologies, Carlsbad, CA).

PCR amplicons were prepared for DNA sequencing with the ExoSAP method using a 10 μl sample of each PCR reaction treated with 5 U of Exonuclease I (New England Biolabs, Ipswich, MA) and 1 U of Shrimp Alkaline Phosphatase (USB) to remove residual primers and deoxynucleoside triphosphate (dNTPs). Bidirectional BigDye Terminator Cycle Sequencing (Life Technologies) reactions of the appropriate template and exon 3 *MYOC* PCR primer were resolved and base called on an Applied Biosystems 3130XL Genetic Analyzer (Life Technologies).

Detection of sequence variants was performed with the Mutation Surveyor v3.10 (SoftGenetics LLC, State College, PA) software; all forward and reverse sequence trace files for overlapping upstream and downstream PCR fragments of exon 3 were assembled by the software into a single contiguous sequence following alignment against the *MYOC* gene GenBank reference NM_000261.1. Significant differences in the relative peak heights of the sequence traces observed between that of the patient sample and a normal control were automatically called a sequence variant by Mutation Surveyor; all such calls were visually inspected for confirmation.

## Results

**Figure 1:**
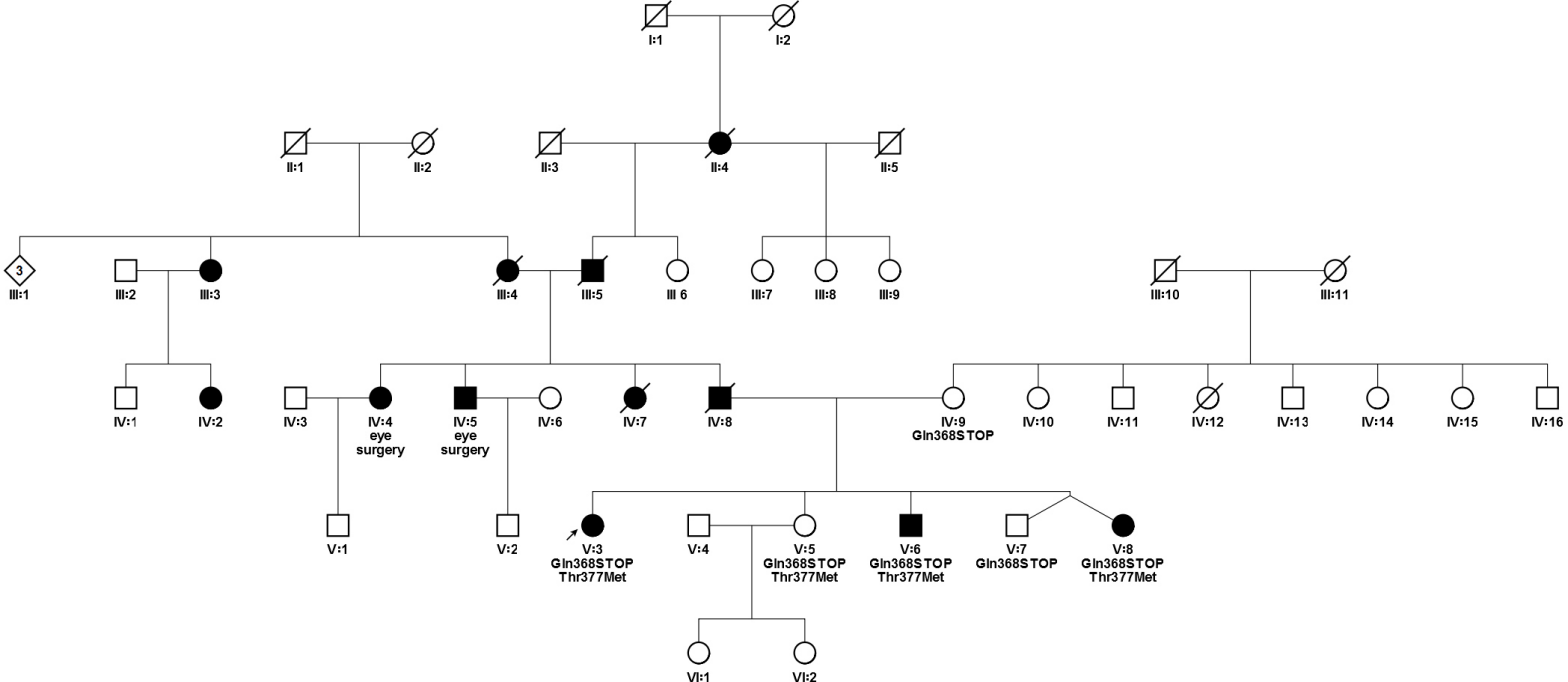
Pedigree chart with Gln368STOP and Thr377Met *MYOC* mutations. Round symbols indicate women; square symbols, men; diamond, gender unspecified; fully filled symbols, primary open-angle glaucoma; unfilled symbols, unaffected; diagonal line, deceased; arrow, proband.

The entire pedigree of this *MYOC* glaucoma family is shown in Figure 1. There are six generations with 46 known members. The family of the proband’s mother (patient IV:9) were Dutch, while the father (patient IV:8) was of Croatian ancestry.

Clinical data of examined family members are summarized in Table 1. All patients had open anterior chamber angles bilaterally with gonioscopy. The average age at diagnosis for individuals with POAG or ocular hypertension was 28 years.

**Table 1:** Summary of known clinical features of the affected family members

Pedigree number	Age	Sex	Age at diagnosis	BCVA		Highest recorded IOP (mmHg)		Centralcorneal thickness (µm)		Visual field defect		Cup/Disc Ratio		IOP treatment	MYOC mutations
				OD	OS	OD	OS	OD	OS	OD	OS	OD	OS		
V:3	35	F	34	6/6	6/6	37	36	569	566	N	N	0.4	0.3	Latanoprost OU	Gln368STOP/Thr377Met
V:5	34	F	-	6/6	6/6	20	18	567	566	N	N	0.4	0.4	-	Gln368STOP/Thr377Met
V:6	29	M	26	6/18	6/5	54	29	582	584	Y	Y	0.5	0.4	3 anti-glaucoma agents OS Trabeculectomy OD age 27	Gln368STOP/Thr377Met
V:7	26	M	-	6/5	6/6	19	19	552	556	N	N	0.2	0.2	-	Gln368STOP
V:8	26	F	25	6/5	6/5	41	37	547	547	N	N	0.4	0.7	Bimatoprost OU	Gln368STOP/Thr377Met
IV:9	63	F	-	6/9	6/9	17	16	566	561	N	N	0.5	0.5	-	Gln368STOP

BCVA: Best Corrected Visual Acuity, IOP: Intraocular Pressure, OD: Right eye, OS: Left eye, OU: Both eyes.

The most severe phenotype is exhibited in patient V:6. This patient presented with a right ischemic central retinal vein occlusion and elevated IOP in both eyes at age 25. He subsequently developed anterior chamber angle neovasularization requiring treatment with indirect scatter laser photocoagulation and intravitreal bevacizumab. The patient’s blood pressure was normal, and physician evaluation for a hypercoagulable state unremarkable. The only identified risk factor for the central retinal vein occlusion was ocular hypertension.

**Figure 2:**
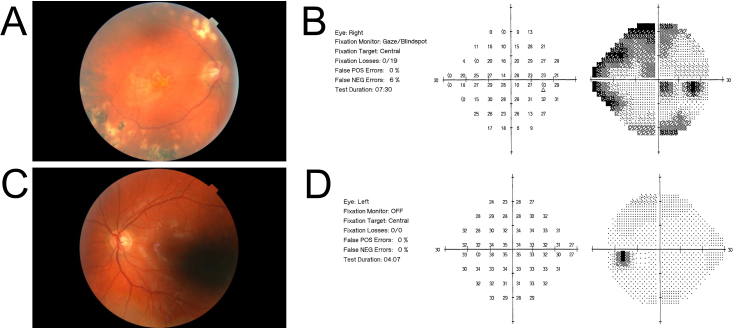
Fundus photographs for Patient V:6. The cup/disc ratio is 0.5 in the right eye (**A**) and 0.4 in the left eye (**B**). Humphrey visual fields for the same patient show a right eye visual field defect due to previous panretinal photocoagulation (**C**).

Fundus examination showed a cup-disc ratio of 0.5 in the right eye and 0.4 in the left with normal neuroretinal rims in both (Figure 2A). Visual field testing was normal in the left eye, but showed an arcuate loss in the right, likely due to the panretinal photocoagulation (Figure 2B).

Two years after his initial treatment, his right IOP became uncontrolled on medical treatment alone, and he proceeded to trabeculectomy with mitomycin C. His IOP is also poorly controlled in the left eye despite the use of three antiglaucoma medications, and glaucoma surgery has been recommended.

The proband’s mother had no evidence of ocular hypertension or glaucoma at age 63. Her seven siblings (ranging from 59 to 73 years old) have refused examination, but are all believed to be unaffected.

The proband’s father was diagnosed with glaucoma at age 45 years (patient IV:8). On this side of the family, nine of 28 individuals were known to have POAG or thought to have glaucoma based on reports of previous eye surgery.

Analysis of the *MYOC* gene in the proband (patient V:3) showed a heterozygous C>T substitution at nucleotide 1102 in exon 3, predicted to generate a nonsense codon in place of the normal glutamine codon at position 368 (Gln368STOP). In addition, a second DNA sequence variant was discovered: a heterozygous C>T substitution at nucleotide 1130 in exon 3, predicted to generate a missense substitution of methionine for threonine normally present at codon 377 (Thr377Met).

Four of five children were carriers of both Gln368STOP and Thr377Met (Figure 1). Of these, three had already been diagnosed with glaucoma or ocular hypertension (ages 25, 26, and 34).

Genotyping could not be performed on the proband’s father as the patient was deceased. The unaffected mother (patient IV:9) was found to carry the Gln368STOP mutation. One of her brothers was screened for the Gln368STOP mutation and does not carry it. The rest of her siblings have not requested genetic testing.

## Discussion

To date, more than 80 disease-causing *MYOC* variants have been identified [3]. Gln368STOP and Thr377Met are the two most common worldwide [4]. We present here the first report of glaucoma-affected individuals carrying both variants. In this pedigree, the proband’s mother (patient IV:9, Figure 1) carries the Gln368STOP mutation alone; therefore, we concluded that the Thr377Met variant was passed down from the proband’s father (patient IV:8) and that children with both would carry mutations on opposing alleles.

The clinical features of *MYOC* glaucoma reflect the underlying mutation. The proband’s father (patient IV:8) was diagnosed with POAG at the age of 45. Glaucoma patients with Thr377Met usually have a disease of intermediate severity with an age at diagnosis of 41.6±13.2 years and a mean maximum IOP of 32.5±10 mmHg [6]. The same mutation has been described in the isolated Croatian village of Veli Brgud, whose population has an unusually high prevalence of early-onset glaucoma [7]. The family of patient IV:8 was of Croatian background and came from a village 10 km from Veli Brgud. It is likely that they share a common genetic basis. Hewitt et al. previously reported that families from Greece, the USA, and Australia, all known to be of Greek or Macedonian ethnicity, shared a common haplotype [6].

The severity of phenotype in Gln368STOP carriers is variable, ranging from ocular hypertension to advanced glaucomatous neuropathy with severe visual field loss [8]. The mean maximum IOP for affected patients carrying Gln368STOP is 29.5±4.5 mmHg, and the mean age at diagnosis is 53±10.5 years [4]. The age at diagnosis may vary considerably; newly diagnosed patients ranging from age 32 to 80 years have been described [4,8]. The proband’s mother carried Gln368STOP alone, and was unaffected at 63 years. As the penetrance at this age lies somewhere between 49% and 98%, this is not surprising [4].

Individuals with both Gln368STOP and Thr377Met appear more likely to develop ocular hypertension or glaucoma from an earlier age than carrying either mutation alone would predict. The three affected individuals carrying both mutations were diagnosed between 25 and 35 years old. The penetrance at 25 years is low for both mutations (3% for Thr377Met and 1% for Gln368STOP) [4]. Juvenile affected carriers of Thr377Met have been described [9]. However, the father carrying Thr377Met in our pedigree was diagnosed with POAG at age 45, so we feel it is less likely Thr377Met al.one is responsible for the young age at diagnosis in the children. Detailed clinical and genotype data for affected members on the father’s side would be of considerable interest, but were unobtainable.

The only patient with both mutations without ocular hypertension was 34 years old at the time of review. It is highly probable she will develop glaucoma in the future, and therefore early treatment was offered and regular reviews scheduled.

To the best of our knowledge, there is only one other report of individuals carrying Thr377Met combined with another *MYOC* variant. In Greece, Thr377Met has been described in three individuals also carrying the Arg76Lys variant [10]. However, Arg76Lys is considered a neutral polymorphism [4], and in the Greek study, several control subjects carried this mutation [10]. Two patients were homozygous for Thr377Met, and demonstrated a more severe glaucoma phenotype than heterozygous cases for this variant [10]. This however was not the case for a patient homozygous for Gln368STOP, who showed no signs of glaucoma at the age of 49 [11].

Currently, the mechanism through which mutant MYOC protein contributes to the pathogenesis of glaucoma is unknown. Gln368STOP and Thr377Met are predicted to change the secondary structure of the MYOC protein [12]. Gln368STOP results in premature termination of protein synthesis [12]. Thr377Met is thought to cause the loss of phosphorylation of the Thr377 site by casein kinase II [10]. Both produce a Triton assay–insoluble protein [12]. The presence of these variants on opposing alleles would predict the assembly of aberrant heterodimeric protein, though further investigation is required to determine why this leads to a more severe phenotype.

Other rare compound heterozygote *MYOC* variants have been associated with glaucoma of earlier onset than predicted by a single mutation. An 11-year-old from Quebec with aggressive juvenile-onset glaucoma was found to be a compound Arg126Trp/Lys423Glu carrier [13]. In addition, a patient from eastern India compound heterozygote for Asn480Lys/Thr353Ile was diagnosed with glaucoma aged 14 years [14], though the Thr353Ile mutation is of uncertain pathogenicity [4].

In summary, we have described the first known pedigree containing compound heterozygotes for the two most common glaucoma-causing *MYOC* mutations, Gln368STOP and Thr377Met. Within this family, both mutations in combination predict a more severe phenotype than either in isolation. Early identification of genetic risk in unaffected family members has strengthened the case for early treatment and monitoring. Our findings will assist clinicians in providing more suitable treatments for affected individuals and appropriate preventive management for unaffected individuals who are compound heterozygotes for these two mutations. Our findings also provide resources for genetic counselors and clinicians to educate patients about their genetic risk profiles.
